# “Dignity as a Small Candle Flame That Doesn’t Go Out!”: An Interpretative Phenomenological Study with Patients Living with Advanced Chronic Obstructive Pulmonary Disease

**DOI:** 10.3390/ijerph192417029

**Published:** 2022-12-18

**Authors:** Carlos Laranjeira, Marília Dourado

**Affiliations:** 1 School of Health Sciences, Polytechnic of Leiria, Campus 2, Morro do Lena–Alto do Vieiro, Apartado 4137, 2411-901 Leiria, Portugal; 2 Centre for Innovative Care and Health Technology (ciTechCare), Polytechnic of Leiria, Rua de Santo André—66–68, Campus 5, 2410-541 Leiria, Portugal; 3 Research in Education and Community Intervention (RECI I&D), Piaget Institute, 3515-776 Viseu, Portugal; 4 Center for Studies and Development of Continuous and Palliative Care (CEDCCP), Faculty of Medicine, University of Coimbra, Azinhaga de Santa Comba, Celas, 3000-548 Coimbra, Portugal; 5 Faculty of Medicine, University of Coimbra, R. Larga, 3004-504 Coimbra, Portugal

**Keywords:** end-of-life care, patients, COPD, dignity, phenomenology, lived experience, home care

## Abstract

Long-term illness, such as chronic obstructive pulmonary disease (COPD), can expose people to existential suffering that threatens their dignity. This qualitative study explored the lived experiences of patients with advanced COPD in relation to dignity. An interpretative phenomenological approach based on lifeworld existentials was conducted to explore and understand the world of the lived experience. Twenty individuals with advanced COPD (GOLD [Global Initiative for Chronic Obstructive Lung Disease] stages III and IV) were selected using a purposive sampling strategy. In-depth interviews were used to collect data, which were then analysed using Van Manen’s phenomenology of practice. The existential experience of dignity was understood, in essence, as “a small candle flame that doesn’t go out!”. Four intertwined constituents illuminated the phenomenon: “Lived body–balancing between sick body and willingness to continue”; “Lived relations–balancing between self-control and belongingness”; “Lived Time–balancing between past, present and a limited future”; and “Lived space–balancing between safe places and non-compassionate places”. This study explains how existential life phenomena are experienced during the final phases of the COPD trajectory and provides ethical awareness of how dignity is lived. More research is needed to investigate innovative approaches to manage complex care in advanced COPD, in order to assist patients in discovering their inner resources to develop and promote dignity.

## 1. Introduction

Chronic obstructive pulmonary disease (COPD) is a progressive lung disease that is a major source of morbidity and mortality and is expected to be the third highest cause of death globally by 2030 [1]. COPD has a significant economic and social impact, which varies greatly depending on socioeconomic status and geographic location [2]. Several risk factors for COPD have been recognized, including accelerated ageing, individual predisposition, and environmental or occupational exposure (e.g., cigarette smoke, air pollution, dust, gases and other irritants) [3,4]. In the early stages, COPD is characterized by the presence of respiratory symptoms such as breathlessness, wheezing, chest tightness and chronic (productive) cough, which worsens during physical activity [5]. Comorbidities arise at more advanced stages of the disease trajectory and include weight loss, which is occasionally associated with cachexia and heart failure, resulting in increased dyspnoea [6]. Depression, psychosocial distress and sleep difficulties are also prevalent and are linked to a lower quality of life and premature death [7,8].

Over the last two decades, there has been an increasing interest in the need and provision of palliative care for COPD patients [9]. One source of concern is whether end-of-life (EoL) care is effectively implemented [8]. According to Braço Forte and Sousa [10], “the EoL of patients with COPD is associated with progressive deterioration, worse quality of life, social isolation and absence of symptom control. The main barriers to a correct and appropriate approach at this stage of the disease are: lack of resources, deficient identification of patients at the end stage and absence of robust studies in the area” (p. e84).

Living with unmet care demands due to COPD disrupts an individual’s existential state, often leading to existential suffering [11]. A growing body of research suggests that existential suffering has a substantial impact on the daily lives of individuals who experience it. COPD care raises challenges, as the disease’s unpredictable trajectory affects patients physically and emotionally, demanding the provision of multiprofessional care to grant comprehensive care [12]. Furthermore, respecting human beings’ rights and preserving dignity are defined as ethical purposes of COPD care.

The Universal Declaration of Human Rights asserts, “all human beings are born free and equal in dignity and rights” [13] (p. 1). Dignity is essential in all human interactions, and is, therefore, a key concept in ethics [14] and the foundation for good EoL care [15]. Dignity is associated with a human being’s ontological and rational nature and corresponds to a universal value that extends to the intrinsic condition of every human being, from conception to death. Human dignity is the anthropological basis for the human rights of every human being. In this sense, dignity is an ethical concept associated with the purpose and intrinsic value of each person, making each person worthy of respect [16]. However, each person’s perception of their own dignity may fluctuate throughout their life cycle. It is through the condition of being rational that autonomy emerges, which, in turn, is the basis of dignity.

According to most scholars, one form of dignity is inherent/inborn and cannot be lessened or changed, but another type of dignity is subjective and changeable and is frequently influenced by external influences [17]. This latter type of dignity is especially important in the context of healthcare, since care delivery can either foster or harm dignity [18,19]. Understanding caring ethics and dignity encourages healthcare workers to take moral responsibility, to be aware of what they see, hear and feel, and to follow an ethical compass [20]. According to Chochinov et al. [21], sustaining a sense of dignity is important for all people in need of care, and upholding these people’s dignity becomes a critical issue for healthcare professionals [22]. Similarly, the sense of dignity has a profound impact on people’s lives and may be a source of personal health and well-being [23].

Several researchers have used qualitative approaches to explore the lived experiences of COPD patients; however, their work focused mostly on physical symptoms and self-care [12,24,25,26] and not on existential difficulties, despite the detrimental impact of existential distress on quality of life [11]. For example, a meta-analysis revealed that people with advanced COPD frequently resort to healthcare, but seldomly engage in dialogues about social and existential concerns [25]. Although dignity has been widely discussed in the literature, there is limited examination of dignity from a first-person perspective. This gap should compel us to consider the depths and details of people’s experiences and their values when elaborating guidelines for care. In this sense, we need to recover the essence of care and discussions of the human experience of suffering and dignity, which are connected to all aspects of an individual’s life [27].

To the best of our knowledge, there is no prior research in Portugal about what patients with COPD think about the concept of dignity. Therefore, there is no information on whether they believe dignity may enhance their care and, if so, how. Because expressions of dignity are influenced by culture [14], the aim of this study was to explore the lived experiences of Portuguese patients with advanced COPD in relation to dignity. The research question guiding this study was as follows: How do lifeworld constituents intertwine with the dignity experience of people who suffer from advanced COPD?

From an inner perspective, we hope to understand personal experiences in relation to dignity and how dignity can be fostered. Knowing how existential experiences (such as dignity) are lived in the latter stages of COPD would allow for more holistic approaches to care in this phase, and thereby, help improve COPD patients’ care.

## 2. Materials and Methods

### 2.1. Study Design

A lifeworld-theory-led, interpretive, phenomenological study was conducted to illustrate that an individual’s reality is always impacted by their surrounding world and cannot be isolated from it [27,28,29]. In this sense, we wanted to investigate the phenomenon by being open to the participant’s life and its significance, in order to improve the practice of professional practitioners. The four existentials that give meaning to the lifeworld and that guided this phenomenological study are: relationality (lived relations), corporeality (lived body), spatiality (lived space) and temporality (lived time) [29]. We advocate that a sensitive and humanized care is grounded in the lifeworld, allowing for the creation of new insights into the human dimensions of care [27].

To “understand the actual character of the object”, this type of inquiry requires the suspension of taken-for-granted beliefs and a scientific attitude, as described by the founder of phenomenology, Edmund Husserl [29]. Although Van Manen provides a systematic framework, he contends that there is no established technique for phenomenology. As suggested by Van Manen [28], we studied participant experiences through a dynamic combination of research activities, such as examining the phenomenon, interviewing, thinking on key themes, and interpreting and developing a description of the event. The identification of themes through data analysis resulted in phenomenological descriptions. The study was conducted and reported according to the Standards for Reporting Qualitative Research [30].

### 2.2. Participants and Recruitment

Participants in a phenomenological research study are often chosen because they have a lived experience with the phenomenon, are eager to share their experience, and can enrich or add to the understanding of a phenomenon’s rich and meaningful experience [31]. Potential participants were identified through electronic records of the pneumology service at a hospital in the central region of Portugal.

Purposive sampling was used, ensuring participants had a notable experience with the phenomenon under investigation. Participants met the following inclusion criteria: (a) adult patients (aged ≥ 18 years); (b) diagnosed with advanced COPD (stage III and IV) according to the Global Initiative for Chronic Obstructive Lung Disease (GOLD) criteria [32], namely, the presence of “clinical symptoms such as increased dyspnoea, acute exacerbations requiring frequent hospitalization, the use of a non-invasive ventilator, and/or long-term oxygen reliance” [33] (p. 3); (c) currently followed in outpatient care; and (d) speak the Portuguese language.

Patients who were cognitively impaired, unaware of their medical condition, and unable to communicate or not keen to participate were excluded. Cognitive screening was completed with the Short Portable Mental Status Questionnaire, which assesses short- and long-term memory and orientation. Each correct answer received 1 point and the global score was the sum of the 10 items, yielding a range from 0 to 10. All participants with ≥ 6 incorrect answers were excluded (moderate cognitive deterioration) [34].

Patients who matched the criteria were sent a letter of invitation with study details and contacted one week later by a telephone call from a nurse. If a participant was interested, an interview was scheduled. Written consent was secured on the day of the interview. None of the patients who were invited to participate in the research declined.

### 2.3. Data Collection

Data were collected between June 2021 and December 2021. The participants chose the venue of the interview. To minimize interruption to their routine, the majority of participants preferred to be interviewed in their own homes. Each patient was given the option of having relatives present.

The in-person phenomenological interviews, covering a wide number of subjects, evolved over the study. Sociodemographic information about the patients was acquired during the interviews. These aspects aided in understanding and contextualizing each patient’s experience. The main researcher also collected field notes to support the information in the recordings (including aspects such as tone of voice, gestures and body position) and noted his reflections about his role during the interview. The aim during the interviews was to listen to participants’ narratives and allow them to talk freely before asking any additional questions, thus demonstrating respect for their experiences rather than imposing the researcher’s preunderstandings. Initially, the participants were somewhat nervous, but throughout the interviews they became more relaxed, making the conversations more fluid. Twenty patients were interviewed, with an average interview length of 40 min.

The first author (C.L.) conducted all interviews. The interviewer had no prior interaction with patients, and interviews were conducted in Portuguese and audio recorded. The interview guide had an approach based on existential experiences. The interviewer sought experientially rich descriptions, emphasized first-person experiences, requested specifics and avoided theoretically loaded questions. To encourage narration, open-ended questions were used, such as “Please tell me what dignity meant to you.” and “How may your dignity be preserved or threatened (related to body, space, time and relational)?” Probing and clarifying questions were asked when needed, such as “Could you please clarify this?”, “What do you mean by that?”, and “Could you please supply me with an example to help us understand your point of view?” The interviews were halted after themes were identified and data saturation was reached. Each patient was interviewed once. Data collection and analysis happened concurrently.

### 2.4. Data Analysis

A phenomenological approach inspired by van Manen’s philosophy [28] was used to analyse the interview texts. Van Manen’s analytical six steps [29] guided the analysis and interpretation of the interviews: “(a) turning to the nature of the lived experience; (b) investigating the experience as we live it; (c) reflecting on the essential themes that illustrate the phenomenon; (d) using the art of writing to truthfully describe the phenomenon; (e) maintaining a reliable and oriented relation to the phenomenon; and (f) balancing the research context by considering parts and whole” [35] (p. 5).

After assembling the findings of the interviews and observations, analytic techniques were applied to discover patterns in the data. Data were coded using an inductive coding approach [36] to produce several codes. Next, codes were grouped. resulting in the development and identification of major themes [36,37]. Lastly, data were used to create a narrative discussion that highlighted the data analysis [37]. At this stage, themes and quotations were translated into English to check for inconsistencies. WebQDA qualitative data analysis software was used to organize the data.

### 2.5. Trustworthiness

The data’s trustworthiness was partly ensured by building trust between the researcher and participants, which allowed the participants to contribute confidential, honest, in-depth information that mirrored their lived experiences of dignity. This study’s trustworthiness is backed by four criteria: credibility, dependability, confirmability and transferability [38]. A study is credible when the researcher’s interpretations correctly represent reality. Evidence is dependable when constant and stable. This was sought through member checks and peer debriefing to guarantee that the interviewer had understood the participant’s meaning. To ensure the dependability of participant experiences, we recorded an audit trail, themes and descriptions. Confirmability is the extent to which data are derived from participant characteristics without researcher bias. This was ensured by documenting all actions, selecting interview quotes to describe findings and generating a study report. To ensure data transferability, study materials were maintained safely, and efforts were made to explain research methods thoroughly, fostering the feasible transferal of findings to other contexts. The purposeful sample, research setting and recruitment methods were explained, and variations in patient experiences were achieved to improve transferability. The data yielded a wealth of information that shed light on the study’s goal, but no new information emerged during the three final interviews.

According to Van Manen [28,29], the preunderstanding should never be overlooked, but rather made explicit. Thus, the research team consisted of an RN working with patients with long-term illnesses (C.L.) and a physician (M.D.), both with extensive experience in palliative care and qualitative research.

### 2.6. Ethical Considerations

The Local Ethics Review Board (approval nº04/2021) approved the study in accordance with the Declaration of Helsinki’s principles. The purpose of the study was explained to all participants, and they were assured of anonymity and an anonymous presentation of the findings. Informed consent was given by all patients. They were told that they might exit the conversation at any time and that there were no right or wrong replies. Recalling vivid memories during the interviews can be unsettling for participants; thus, our main priority was the comfort and well-being of the participants. At the start of the interview, participants were told that the interview might be paused or terminated at any point without explanation.

## 3. Results

### 3.1. Sample Description

A total of 20 participants were interviewed. Most of them were male (n = 15), with an age range between 58–82 years (M = 66.85; SD = 7.21). The time since CPOD diagnosis ranged from 4 to 11 years (M = 5.9; SD = 2.04), and the majority were in stage III (n = 14). Fifteen participants were retired. More than half of the participants were married (n = 15) and had received a secondary education (n = 12).

All the participants used noninvasive ventilation, nebulisers and oxygen therapy as a means of treating COPD and stated that dyspnoea was disabling and one of the main struggles of having COPD. Most participants had a history of smoking (n = 16) and, of these, only 6 had stopped smoking. The majority reported being retired or unemployed due to limits imposed by their illness. No participants accessed specialist palliative care at the time of the interview, receiving only support from community-based services. Details about participants are depicted in Table 1.

### 3.2. Findings from Interviews

**Figure 1:**
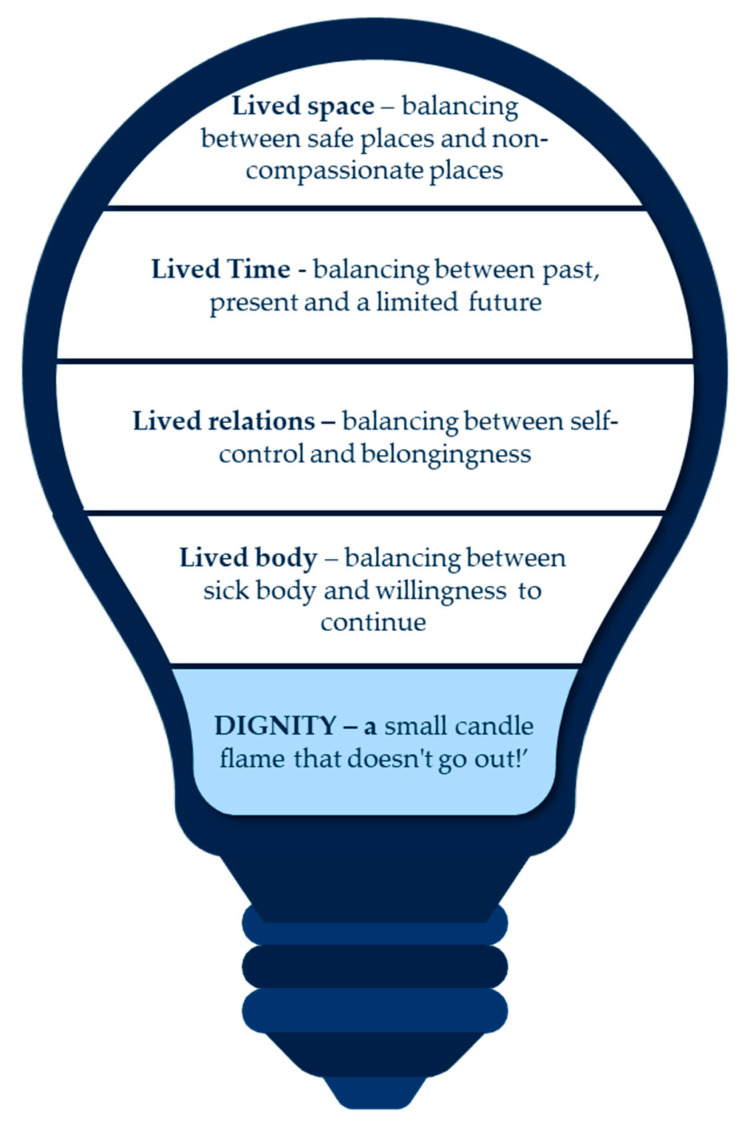
Conceptual model of the phenomenon of lived experiences of advanced COPD in relation to dignity.

The existential experience of dignity was understood, in essence, as “a small candle flame that doesn’t go out!”. In the following, we elaborate on how the lived experience of dignity comprised four interrelated themes: “Lived body–balancing between sick body and willingness to continue”; “Lived relations–balancing between self-control and belongingness”; “Lived Time–balancing between past, present and a limited future” and “Lived space–balancing between safe places and non-compassionate places”. These four themes were described in the present time, clarifying the phenomenon’s fluctuation in time and space, body, and relational experience (see Figure 1). The study themes and quotations from participants are described below.

As their illness took over their daily lives, some individuals felt more distanced from their own selves, occasionally responding in unexpected and unpleasant ways. They recounted being emotionally and existentially disturbed, as serenity and harmony were replaced with sorrow and doubt: “I feel a lot… not exactly anxiety, but something like that… it’s a whirlwind of feelings that threatens what I am, my dignity. The problem with showing this is that I feel guilty and frustrated… I cry a lot, and I have no problem with it… but I feel like I’m doing something wrong…” (P10); “In moments of greater instability, I feel that I am no longer me, but then when I calm down, everything becomes easier… dignity is like a small candle flame that despite the strong wind doesn’t go out… it can get weaker, but it continues to shine” (P5).

#### 3.2.1. Lived Body—Balancing between Sick Body and Willingness to Continue

The term “lived body” refers to our physical body or bodily presence in our daily lives, which includes all we experience, expose, hide and communicate through our lived body. When breathing occurs smoothly, it generates a pre-reflective essence of embodiment. Breathing as a finite number of breaths and breath-taking moments is defined as an often unconscious and forgotten truth of existence. Only when breathing becomes difficult, and one gasps for oxygen, does one realize that breathing is a necessary requirement for any action and for life in general. Breathing was an ever-present and explicit activity for the participants in this study. Breathing assumed a core action around which daily life was organized. The unpredictability of dyspnoea was clearly one of the greatest burdens for several participants.

P3: “Shortness of breath is unpredictable, it appears every three or four weeks–it feels like I’m drowning, it’s horrible. The tiredness and anxiety are terrible… my body is exhausted, and in those moments, I think I’m going to die.”

For some participants, dignity becomes difficult to achieve in ways each patient recognizes themselves, due to the limitations the sick body imposes. In daily care, participants perceived the unpredictability of symptoms and their inability to manage daily activities. Patients described suffering because their bodies are unable to respond to daily requests. The body has no strength anymore, and surrenders itself to the disease.

P1: “The despair of being able to breathe and not being able to, generates a frustration that limits me. I think about what I was, the energetic person who did everything independently. And now [pause]… I feel trapped in my body, it doesn’t obey my will!”

P1 revealed that he is familiar with his body, as he realizes that maintaining his previous active life can put him at risk, given his intolerance to exertion and propensity to develop a dyspnoea crisis. Losses and limitations are felt as progressively worsening. The participants are aware that their bodies are failing. They also understand that if they are inactive, the downturn will accelerate. Participants strive hard to keep their bodies in shape and avoid losing muscle mass out of need and self-protection. Participants’ lives are dominated by their concern for their own bodies. Rather than viewing this as negative, some see it as giving them a purpose in life and contributing to their well-being, as it allows them to organize their day and generates a nice feeling in the short term, even though the entire endeavour is perceived as a losing struggle.

P4: “My legs hurt; my muscles are stuck. Again and again, my body limits me. But I stay active until I can’t do anything else. Because if I don’t, my life will lose its meaning.”

P8: “Currently I have difficulty doing activities that I used to do without any problem… I feel a slowdown in all my activities, and this affects me a lot.”

An unavoidable relapse or hospitalization that prohibits or restricts moving for an extended period may mean the hard-fought physical condition is soon lost. The deterioration in lung function is overwhelming, since COPD is stronger than the body and the will. The participants understood that no amount of excellent behaviour would make a difference. Even if their body failed them, their motivation and confidence continued. P15 said: “My body breaks, my legs are weak and that limits me a lot, but having kept my strength and motivation, I can’t give up”. P18 said: “Every time the symptoms reappear, I feel that my body weakens, it is harder for me to recover… but I maintain confidence that I will make it”.

#### 3.2.2. Lived Relations—Balancing between Self-Control and Belongingness

The human interactions with others within a shared interpersonal space are referred to as lived human relations. Participants reported feeling “labelled” by their illness, which challenges their sense of “self” and “identity”: “From the beginning of the disease I knew that it was a chronic situation… In fact, I was making plans for my life, hoping to get better, but now I know that death is near, so I… I want to understand what is going on, but there is a part of me that is terrified. I’m afraid of feeling an agonizing pain that robs me of who I am” (P2).

The participants strongly desired to make choices and have control over crucial decisions in their life, as this helped them retain their self-esteem and autonomy. Feelings of control were heightened when others boosted their perceptions of independence and autonomy. This was perceived as courteous, helpful and compassionate—an acknowledgement of their right to choose while confronting their EoL: “I already told my daughter that… when I can no longer speak, I want to let myself die. Having dignity is just that, being able to choose what I want and what I don’t want. And feel that those around me understand this and accept my decision” (P1).

While the hospital setting generally offered a secure and supportive environment, when transitioning to home care some patients felt “alone” and less supported: “…when you go home, you’re very much on your own…now I need a little aid and support… I have the impression that I am receiving a poor…well, not a poor service, but a restricted service” (P8). The most common fear was that they would become a burden to others, particularly family members, resulting in a loss of dignity as a result of their incapacity to care for themselves. P14 states that: “My biggest fear is overloading my children, life is not easy… my pension is small, and I can’t cover all the expenses, my children help me, but that’s really hard for me”. P11 also stated: “With my illness, I had to retire at the age of 50, since then my wife has had to work hard so that we don’t lack anything at home. I get angry about it and sometimes we even argue about it, but she understands me.”

Some participants described feelings of grief when deprived of a sense of control over life, due to physical weakness and their psychological needs. Feeling vulnerable, patients described some situations created by health professionals where their autonomy was threatened and they experienced a loss of power and independence. As P2 said, “The professionals are very kind… and always available. But sometimes I feel like they pity me… when I’m most incapacitated and I need help bathing, they do everything. I even tell them that I want to help, but they [professionals] won’t let me. In these moments I feel that I am worthless, my privacy is disrespected… it is not worth living like this”; and P19: “They mean no harm, I know! But sometimes, I feel that I can do it for myself, it may take longer, but I can… it’s just that the nurses, as they have a lot to do in a short time, end up doing it for me. This happens during meals, in the shower or when getting up to the highchair”.

P13 described feeling ashamed and stigmatised by healthcare professionals: “They look at me like there’s something wrong with me. Coughing, shortness of breath and oxygen equipment make me feel like a stranger. The doctor told me that I’m like this because I smoked two packs a day. I’ve always been a smoker, but I don’t think it was just that… maybe I’m just unlucky. Others also smoke and do not have this problem.”

Respect for others was essential throughout the decision-making process. During interactions with professionals, some participants mentioned the importance of feeling respected and valued, and of being treated as a human being. Having fruitful relationships is essential for creating meaning. Positive communication focused on the sick person’s needs seemed to contribute to promoting dignity and positive relationships between professionals and patients. In this regard, P2 said: “In moments of greater difficulty, it is so good to be able to count on the support of those who take care of us, we feel supported and above all respected”; and P7: “I think that dignity depends a lot on the recognition that others have of me, that I still have value after all.”

When explaining the impact of their illness, all individuals seemed to echo similar opinions. Most thoughts were of loss: loss of employment due to early medical retirement, loss of family and social contacts and, for P9, loss of intimacy. Nonetheless, patients sought to devise coping strategies that took their limitations into account. Feeling loved appears to have had healing effects, being capable of transforming feelings of devastation into strength and hope. Love was important to the participants’ existence. They realized deep love within themselves, which enabled them to withstand hardship. P16 and P20 both stated that expressing love gave them the strength to stay alive.

P16: ”the disease allowed me to strengthen ties in my family! I had an older brother with whom I didn’t have much connection, in fact, we had been apart for years, and since he found out I was worse he came to see me! I’m very happy…”

P20: “there is one thing that the disease brought, it was family unity, my grandchildren see my condition and are always available for whatever I need. Just last week they took me for a walk, I went to see the sea… (crying), I couldn’t remember that feeling anymore.”

#### 3.2.3. Lived Time—Balancing between Past, Present and a Limited Future

The notion of lived time differs from our experience of clock time or objective time and is related to our temporal manner of being in the world. Participants felt the walls of time closing in and, eventually, the sense of lived time reducing from big and expansive to small and constrained, akin to the “matryoshka doll,” which depicts a multilayered person.

P2: “I feel like a matryoshka, as time passes, I feel smaller, more compressed with the clear certainty that many of my desires will not be possible to come true… in the most difficult moments I resort to prayer to find some strength to carry on!”

Their approach regarding time was different, having learned to focus on the spiritual element of their life. Spirituality, as an act of expressing and seeking meaning and connecting to a higher force, can be of valuable assistance in adjusting to one’s own EoL situation. After deepening spiritual issues, people reported a higher feeling of well-being and self-determination, allowing them to accept death into their life. In this sense, some participants declared a transcendent horizon of time, where death was not an end but the beginning of a new life without worries.

P10: “If I die today, I will have the opportunity to live again… I have the impression that God is around me… I know I may confess my transgressions to him, and that he will forgive me. I can tell you everything since he’s a terrific buddy I can rely on.”

P18: “God… give me strength… I have no fears.”

The sense of struggle was central to many patient accounts, as they sought to find meaning and hope in their lives in the face of an unclear and shifting future with a life-limiting illness. Wishes and expectations were portrayed via feelings of hope and resilience when vulnerable. “When you don’t know what’s ahead of you… I want to gain the strength to get through it. This is important to me… and to those around me. I want to be able to die in peace… with dignity!” (P20).

P10: “I’m never alone, I have faith and hope… I think that tomorrow I’ll be a little better.”

Patients perceived their past as more dominant compared to their future, arguing that this is so because they have limited life remaining. They preferred to reminisce about happy memories from their past. The analysis also revealed that being anchored in the present while, at the same time, rescuing memories of the past made it possible to dream and sustain hope, creating some sense of time. “The future will bring death… after all, it comes to everyone. But I just don’t know how it’s going to be, how…? You know, I had a good life, I was loved, my children are raised, so right now I hold on to the memories that remain. It’s these memories that keep me fighting, and dreaming that even if I’m not there, they’ll be fine!” (P12).

#### 3.2.4. Lived Space—Balancing between Safe Places and Non-Compassionate Places

The lived space is where humans move and feel at ease. Having a location to call home and a safe space to escape to and return to symbolizes human mobility in the world. Having a safe place expands the lived space. On the contrary, being robbed of one’s own protected zone disrupts the illusion of living in free space.

Most participants recognized the relevance of the home space, which they described as a safe place to be and a space of companionship and support, and “natural” settings, where there should be caring, dedication, warmth and attention. However, several aspects can influence the experience at home, particularly the progression of the disease and the need for support from close relatives. All participants emphasized the importance of spending their last days at home, naming it as a place for a “good death”, surrounded by love and, above all, good memories. Thus, the findings suggest a strong relationship between the participants’ lived space experience and their need to retain feelings of belonging, purpose, security and autonomy. These were some of the qualities that contributed to the capacity to maintain a sense of self-identity, preservation and attachment to a place.

P6: “My house is all I have, I helped build it and that makes it a part of me. So, when I die, I want to be here… surrounded by those who love me; P9: When I get up in the morning, I look out my window and I see my backyard, the trees I’ve planted, and I think how good it is to be here.”

They experienced “home” as promoting autonomy, allowing them to care for themselves and use resources such as assistive technology (e.g., ventilator and oxygen therapy). They expected to use assistive technology because it plays a vital role in adapting to a new life situation. The loss of previous and valued activities, such as the ability to drive, was connected to existential aspects and grief over lost activities and roles. When an individual’s body becomes practically immobile owing to fatigue and a loss of muscle mass, their reality becomes confined and altered. This new reality is characterized by a tighter living room and space, both perceptually and symbolically, which will stay constrained for the rest of the individual’s life.

Contact with nature was also mentioned as relevant, although this contact was limited by fatigue. The use of support equipment, such as a wheelchair, can minimize this impact. “Walking in the garden at home” (P13), “feeling the fresh air“(P4) and “sunbathing on the balcony” (P9) are examples that helped patients maintain a sense of well-being when facing their current status, and thus, promoted personal dignity. In turn, being “disconnected” from home could undermine patient identity, as the home is a deep component of identity and a location where identities are expressed. At this point, paying attention to the various meanings of home becomes critical, since it represents an environment where the patient may express their particular identity.

Several participants in this study shared similar stories. Whether at home or in healthcare facilities, some participants reported being cared for with compassionate care versus non-compassionate attitudes and limited support from professionals. Such attitudes were reflected in pleasant and stimulating contextual experiences or, on the contrary, in mechanized environments, decentred from individual needs. They wanted to be seen as a whole person, rather than feeling like their healthcare professionals were focusing on medical matters alone. P5: “In the hospital, everything is different, when I’m worse and I go to the emergency room, I get even worse. The noise, the closed environment, the lights on all night. Anyway, I get very anxious and that doesn’t help me at all… When they send me to the pulmonology inpatient ward, things get a little better. Nurses are concerned with creating a more pleasant environment”; and P15: “When I was hospitalized, they [professionals] did what they could… there’s a lot of work, but the lack of staff doesn’t help either. They do a lot!”

The patients also expressed ambivalence associated with the home when this environment took on hospital-like characteristics. One participant (P17) said: “My house is different from what it used to be, my room looks like a medical ward, it is the ventilator, the oxygen bottle, the articulated bed, the wheelchair… My wife works hard to make the environment pleasant, but when I look around, everything is different… but then I think what matters is having the people I care about with me, that’s being at home!*”* As this participant explained, when he requires care, his perception of home and his feelings change. However, the home’s value is in “having the right people around you”, not its physical constituents, because being at home is being connected with loved ones.

## 4. Discussion

Anchored in Van Manen’s phenomenology of practice, this study explored the lived experiences of patients suffering from advanced COPD in terms of dignity, providing novel insights into the priority topic of end-of-life care. Our findings are consistent with those outlined in the literature, namely, a universal sense of a vulnerable and disrupted body determining a patient’s entire life [39,40]. The disruption of the lived body was felt as a breakdown in the biological body’s mechanical functioning, expressed by breathlessness and exhaustion, but also as a disruption of everyday activities around which life is organised [40]. The loss of autonomy or control over the body was frequently perceived as a loss of one’s own identity, with profound effects upon the critical sense of dignity [41]. Despite this, paradoxically, individuals tried to maintain motivation as a reaction of the embodied subject to retain their wholeness and dignity.

The relationships along the body–others–world axis are profoundly impaired by the patient’s physical incapacity. Qualitative studies show that any shift in the lived body can affect the individual perception of dignity [19,41,42,43]. However, as Rodríguez-Prat and Escribano [41] highlight, “while the loss of functionality may be one factor that leads patients to experience their illness from the viewpoint of the objective, biological, or functional body, the recognition of personal resources (e.g., confidence and motivation) and understanding by others of their symptoms and suffering could help to generate new attitudes and strategies for coping with illness from a more positive perspective” [41] (p. 293). The patient’s experience may also be improved if they feel others have a better understanding of what it means to live with an advanced illness [44]. An active sense of motivation fosters patients to adjust to the trajectory of the illness and helps them to feel secure [45].

As a counterpoint, evidence supports the notion that patients who experience a loss of sense of continuity, as well as diminished motivation and confidence in projecting themselves into the future, are more likely to relive previous unsuccessful or frustrating experiences and, as a result, are more vulnerable to suffering from demoralization syndrome (defined clinically as feelings of hopelessness and helplessness induced by a loss of purpose and meaning in life, and identified by a lack of drive or motivation to cope) [46,47]. Furthermore, evidence shows that demoralization is common in progressive illnesses and is strongly linked with persistent physical issues, a lower quality of life, and psychological problems such as depression, anxiety and a wish to die sooner [47].

Another crucial element is the perception of dignity mediated by the experience of autonomy or control [41] in terms of relations with others. As a result, depending on the nature of the relationships, one’s sense of dignity might be violated, reconciled or even increased. Likewise, interpersonal connections can either produce or endanger existential significance. As current studies demonstrate, patient preferences and autonomy must be respected, as patients are the experts in their own lives. Gómez-Vserda et al. [48] underlined the relational character of human beings and our fundamental need for support from others in order to exist. Inasmuch as COPD, in many circumstances, leads to full dependency on others, it threatens the sense of control and autonomy. Despite the distressing experience of illness, some studies report that an awareness of vulnerability represented an opportunity to re-establish ties with loved ones, promote union with others through reconciliation and forgiveness [49,50], and see one’s final moments as an opportunity to transmit values (legacy) to other family members [49,51]. This evidence is similar to our findings, as some patients recognized family ties as indispensable for their functioning.

The ongoing nature of chronic illness imposes a significant strain on both patients and family members. Responsibilities within the family can change because of the illness, which can cause problems and disrupt the peace of family connections. Some participants had to retire early due to their deteriorating health, having to rely on their spouses and family members to help with domestic chores and shopping. A loss of income may cause financial difficulties and place more anxiety and pressure on the family [52]. These shifting responsibilities and changing circumstances may cause misunderstandings or even conflict within the family [53].

Our findings also suggest that illness affects how participants see themselves and others (family, friends, healthcare professionals and the immediate social environment), as well as how social and professional responsibilities shift [54]. The “clinical gaze can sometimes become ‘objectifying’, in the sense that it is unable to comprehend this subjective, personal world, which needs a human and humanizing attitude to help mitigate the feeling of vulnerability” [41] (p. 290). A previous study found that good communication is an important component of a patient-centred model of care for helping patients become active agents in their own healthcare [55,56]. Nevertheless, if the connection is strained, it may be difficult for professionals to provide individualized care. A diagnosis in medical culture can also imply judgment on one’s way of life. Studies indicate that some COPD patients feel embarrassed and stigmatized about their diagnosis since healthcare personnel state that they are to blame for their condition [57,58].

As the participants’ lived experiences demonstrate, the illness changed their meaning of past, present and future, and their sense of time and rhythm [59], regarding their period of illness and their overall biographical time (“life”). This affected their acceptance of the condition as a “way of life” rather than an “illness” [59]. Some studies have begun to explore the sense of time for the chronically ill and its effects upon the roles they perform [60], how they approach daily activities, and how they constantly renegotiate their identity and illness’ influence on how time is experienced [60,61].

Our analysis also demonstrates how participants reflected on the meanings attributed to past memories, bringing them into the present and projecting them into the future, while sometimes rooting themselves in the past to help maintain their personhood. Spiritual beliefs were deeply ingrained in their lives and were obviously part of how they described themselves (personal identity) and their reasons for feeling hopeful and comforted. Their faith undoubtedly provided them with the fortitude to deal with what was happening to them [62]. Respect, spiritual peace and hope have been identified as key attributes of dignity in the context of dying [63], and actions that can conserve dignity in this context include seeking spiritual comfort, finding solace within one’s religious or spiritual beliefs, and fostering feelings of hopefulness [22].

The findings show a clear link between lived space experience and the need to maintain experiences of belonging, meaningfulness, safety and security, and autonomy in people with advanced COPD [22]. Participants described the home environment as a place for safety and security, an arena where they assert control of their bodies and emotions, avoid stress, do things at their own pace, and where the abilities of caregivers can be improved to meet their needs and wishes [64].

Interestingly, and in keeping with the findings from our study, some studies describe how being outdoors in nature or a garden might have positive consequences, including by fostering emotional well-being and feelings of freedom, and by preventing loneliness. Exposure to nature, particularly green and blue spaces, has been linked to a variety of health advantages, including mental health benefits [65], better psychological well-being [66] and the regulation of body rhythms [67].

In our study, the home appeared as “a place for a good death”, which in the literature represents a common measure of quality of EoL care [68]. Dying at home is, therefore, envisioned as a reply to the growing imperative to preserve patient dignity and autonomy, while also addressing the need of healthcare systems to save money and lessen their load on secondary care supplies [69]. Similarly, compassionate spaces in healthcare are regarded as a standard of care and a critical component of a patient’s healthcare experience, but availability is insufficient. As per the latest studies, compassion is built into healthcare settings, but it can be altered by life events and can change over time [70]. A recent scoping assessment confirmed the limitations of compassionate care as mentioned by participants, highlighting a range of barriers to compassion within practice settings, such as time constraints, workloads and staff shortages [71].

The fragmentation of healthcare settings or the lack of compassion, mentioned during interviews, can be overcome with a more structured care network, including palliative care services and multiprofessional care that properly manages the multiple dimensions of complex care for patients with advanced COPD [71]. Although COPD is recognized as a life-limiting illness requiring palliative care, such care for this group is lacking in Portugal [1]. In this sense, our study confirms this scenario, since none of the participants benefited from an integrated palliative care plan. Besides the scarcity of home-based palliative care services, other reasons could explain this reality, namely, the unpredictable trajectory of COPD and the misunderstanding of palliative care as being exclusively for patients with cancer and only useful in the latter days of life [72,73,74,75,76].

Maintaining patient dignity is the duty of the healthcare provider, but also of the patient’s family and the patient themself. Additionally, policymakers and leaders in healthcare play an important role in promoting healthcare that respects people’s dignity. From the stories and our interpretations, there is an appeal to the ethics of the face proposed by Emmanuel Levinas [77], where responsibility should be a prerogative in the face of the other affected by vulnerability and suffering.

### 4.1. Study Limitations

There are certain limitations to this study that must be noted. First, the findings, such as those of previous qualitative studies, cannot be generalized to the target population. However, there are broad, phenomenological insights into how individuals with COPD perceive the important, universal and recurring features of their experience of dignity. Second, the participants in this study were chosen using a purposive selection method. The number of participants was deemed sufficient to achieve variance and maintain depth in the study. After 17 interviews, data saturation was reached; nonetheless, three extra interviews were conducted to ensure that further coding was not possible and that no new themes were identified. Although results were based on a single interview per patient, the material was sufficiently substantial for the researchers to reach a shared interpretation of the data. In addition, several strategies were used by the researchers to reduce the possibility of biased judgements and idiosyncratic interpretations. Further research should investigate the influence of culture on dignity experiences in other groups of advanced ill patients to increase the transferability of findings.

The recruitment process produced a sample of participants who were not receiving palliative care. This could imply a lesser understanding of the role palliative care may play in advanced COPD as compared to that from a more diverse sample of respondents.

Furthermore, specific participant characteristics were not evaluated, such as economic status or religious beliefs. Restrictions associated with the COVID-19 pandemic hampered both the recruitment and interviewing processes. Nonetheless, with the support of partners and additional precautions, the project persisted, and the findings were not affected by the pandemic conditions.

### 4.2. Implications for Practice

The findings of this study have important clinical and research implications. Assisting patients with advanced chronic illness to live and believe in their ability to live while ill is a critical component of comprehensive and integrative care. Lifeworld existentials provide researchers with a method of inquiry that is congruent with humanistic practices and that acknowledges the uniqueness of the individual within the world [78].

Confronting existential concerns about life and death, which can have positive effects, should be included in professional and informal support systems. In addition, access to social and healthcare services, which presently tend to be fragmented, needs to be addressed. We encourage healthcare providers to conduct these discussions with patients and families in order to better foresee their individual needs. Further research should concentrate on developing, implementing and evaluating supportive interventions aimed at improving people’s psychosocial and spiritual well-being as they approach death. Given that many factors impact the sense of dignity, making care plans informed by a phenomenological stance would help defend the dignity of patients (and their families), thereby ensuring a higher quality of life. Additional qualitative research is needed to better understand the significance and value of dignity as a health resource in people’s lives, and how boosting dignity might help individuals feel better. Finally, we must improve our understanding of the provision of palliative care in advanced COPD and how these services can be implemented successfully.

## 5. Conclusions

To summarize, this study provided an innovative contribution to our understanding of the relevance of dignity perception in the lives of advanced COPD patients. Patients needed to adopt this principle in their lives which will have a positive impact on their well-being. Our findings illustrate a set of holistic issues that had an impact on participants’ dignity needs, including four closely intertwined constituents: “Lived body–balancing between sick body and willingness to continue”; “Lived relations–balancing between self-control and belongingness”; “Lived Time–balancing between past, present and a limited future”; and “Lived space–balancing between safe places and non-compassionate places”.

This research showed that exploring the notion of dignity via patient discourses may help acquire knowledge about dignity from an inner viewpoint, a knowledge that healthcare professionals and educators should integrate into their clinical and educational practices. Because dignity is frequently endangered when patients are very sick, professionals must support and strengthen patients to live and relieve their suffering so they can be themselves and feel dignity. Similarly, as revealed in this study, it is critical to help patients discover their inner resources that develop and promote dignity, including elements such as a “purpose in life” and “love as a restorative energy”. More research is needed to investigate innovative approaches to manage complex care in advanced COPD and to clarify how palliative care can fit into this complex care network.

The flux of demands in COPD, as illustrated in this phenomenological study, requires service support and flexibility so that both professionals and patients can adjust to the disease’s unanticipated yet rising demands over time. This requires an interdisciplinary approach, incorporating health and social fields.

## Figures and Tables

**Table 1:** Sociodemographic and clinical data of the participants (n = 20).

Participant	Age (Years)	Sex	Marital Status	Educational Level	Job Status	Time Since Diagnosis (Years)	GOLD Class *
P1	63	Male	Married	Secondary education	Retired	4	Stage III
P2	59	Male	Divorced	Secondary education	Unemployed	8	Stage IV
P3	66	Female	Married	Secondary education	Retired	4.5	Stage III
P4	63	Male	Married	Secondary education	Unemployed	4	Stage III
P5	59	Male	Widowed	Higher education	Unemployed	4	Stage III
P6	71	Male	Divorced	Secondary education	Retired	5	Stage III
P7	74	Male	Married	Basic education	Retired	6	Stage III
P8	70	Female	Married	Secondary education	Retired	4.5	Stage III
P9	68	Male	Married	Higher education	Retired	7	Stage III
P10	58	Male	Single	Secondary education	Unemployed	4.5	Stage III
P11	65	Male	Married	Secondary education	Retired	5	Stage III
P12	58	Male	Married	Higher education	Unemployed	8	Stage IV
P13	62	Male	Married	Secondary education	Retired	5.5	Stage IV
P14	70	Male	Married	Secondary education	Retired	8	Stage IV
P15	73	Female	Married	Basic education	Retired	3.5	Stage III
P16	62	Female	Married	Higher education	Retired	9	Stage IV
P17	82	Male	Widowed	Basic education	Retired	6	Stage III
P18	74	Male	Married	Basic education	Retired	4	Stage III
P19	60	Male	Married	Secondary education	Retired	7	Stage III
P20	80	Female	Married	Secondary education	Retired	11	Stage IV

* Clinical criteria: stage III (severe) or stage IV (very severe) based on their level of airflow limitation (Global Initiative for Chronic Obstructive Lung Disease–GOLD [32]).

## Data Availability

All data generated or analysed during this study are included in this article.
